# Etiological Anomalies in Cholera

**Published:** 1854-09

**Authors:** 


*Etiological Anomalies in Cholera.*—The following extracts from letters just received from a friend, seem worthy of permanent record as undoubted and very remarkable facts in the history of cholera. We shall, however, draw no inferences from them, but leave them as themes for the exercise of the ingenuity of those who take different sides in the question of the causes of cholera. It is, at least, curious to find, exclusively exempt from the malady, the very places which, according to the views generally prevalent, ought to be its especial haunts. We do not intend, in conformity with the playful suggestion of our non-Medical Correspondent, to get a Black Hole made to test the preventive powers of foul air and crowding; but we doubt not the facts here recorded will be regarded as strengthening the arguments in favor of one much-disputed cause of the malady:—

I.

"*Mexico*, July 2, 1854.

"Here we are infested with cholera, which has been with us since November last; but it has of late been very severe. The cases are most violent—much more so than in its former visits, and attacks the higher classes rather than the lower. It is a singular thing that there has not been a single case in any of the prisons. You have seen the Tepic and Guadaloupe prisons. Bad and filthy as they are, the 'Acordada' and the 'Diputacion' of this city are much worse,—the filth, putrefaction, and stench are beyond all conception; and yet, while people in elegant bed-rooms are carried off in five or six hours, not a case has occurred in those filthy nests. You may recollect we made the same observation in Tepic in 1833. This is worth studying."

II.

"I have received the Mexican letter, and return it to show you the extraordinary circumstance of people not dying of cholera when shut up in close, filthy prisons in Mexico. I well remember that in 1833, the prison of Tepic (in Mexico) was entirely exempt from cholera, when the rest of the population lost about one-tenth. This, at the time, I thought might be owing to some accidental cause; but it seems to be a general result. This prison of Tepic was beyond anything you could have seen in your day. It was filled often to cramming, and had only one small window, or rather air-hole, to ventilate a place containing 250 people! This hole was placed just below the roof or ceiling, and admitted very little light. I had the temerity to go into this prison to measure its capacity, but I was very glad to get out of it. Yet this black-hole, in the time of a very severe cholera, lost no more than its ordinary numbers in ordinary times; I even think less! Can you scientific men account for this? or could this fact be of any use in suggesting a remedy or preventive for this terrible disease? Do get a black-hole made at some place where there may be cholera, and make the experiment?"—*London Medical Times and Gazette.*

